# Altered gut microbiota correlated with systemic inflammation in children with Kawasaki disease

**DOI:** 10.1038/s41598-020-71371-6

**Published:** 2020-09-03

**Authors:** Jie Chen, Yanghua Yue, Lu Wang, Zhonghua Deng, Yonghua Yuan, Menghua Zhao, Zijie Yuan, Chaochao Tan, Youde Cao

**Affiliations:** 1 Department of Laboratory Medical, The First Affiliated Hospital of Hunan Normal University, Hunan Provincial People’s Hospital, Hunan Normal University, Changsha, 410005 China; 2 Department of Pediatrics, The First Affiliated Hospital of Hunan Normal University, Hunan Provincial People’s Hospital, Hunan Normal University, Changsha, 410005 China

**Keywords:** Microbial communities, Inflammatory diseases

## Abstract

Kawasaki disease (KD) is a multi-systemic vasculitis of unknown etiology that occurs mainly in children, and the disturbance of gut microbiota is generally believed to cause a hyperimmune reaction triggering KD. The aim of the study was to investigate the alterations in the fecal microbiota and assess its relationship with systemic inflammation. Totally 30 KD children were enrolled and followed up for 6 months, with another group of 30 age- and sex-matched healthy children as controls. Phylotype profiles of fecal microbial communities were analyzed using 16S rRNA gene sequencing. Serum inflammatory markers were detected by flow cytometer. We showed that KD children exhibited a significant reduction in fecal microbial diversity in the acute phase compared with the healthy controls. *Enterococcus*, *Acinetobacter*, *Helicobacter*, *Lactococcus*, *Staphylococcus* and *Butyricimonas* in acute KD children were significantly higher than the healthy children. Levels of systemic inflammation biomarkers, including IL-2, IL-4, IL-6, IL-10, TNF-α, and INF-γ, were significantly elevated in the acute KD children. Altered microbiota genera *Enterococcus* and *Helicobacter* abundances were shown to be correlated positively with IL-6, which were never previously reported in KD. This study suggested that gut microbiota alteration is closely associated with systemic inflammation, which provides a new perspective on the etiology and pathogenesis of KD.

## Introduction

Kawasaki disease (KD) is an acute febrile disease characterized by multi-systemic vasculitis that primarily invades the small and medium-sized muscular arteries, especially for the coronary arteries^1,2^. Currently, KD is considered the leading cause of childhood-acquired heart disease in developed countries^3^. Moreover, KD is also a risk factor for cardiac disease and other pathogenic conditions when children grow older^4^. However, the etiology of KD still remains poorly understood. The disturbance of gut microbiota has been revealed to induce several diseases including inflammatory bowel disease, allergic diseases, and autoimmune diseases^5–7^. It is also thought to induce a hyperimmune reaction and trigger KD in genetically predisposed children^8^.

Cumulative evidences suggest that the perturbation in gut microbiota composition is closely associated with KD pathogenesis. Specifically, the proportion of potentially harmful HSP60-producing gram-negative bacteria including *Neisseria*, *Acinetobacter*, *Enterobacter*, and *Veillonella*, and the presence of gram-positive cocci with superantigenic properties including *Streptococcus* and *Staphylococcus* are elevated in KD children compared with healthy controls^9,10^. A higher incidence of potentially harmful *Eubacterium* and *Peptostreptococcus* is also observed in KD children than in children with other febrile diseases^11^. Moreover, the incidence of *Lactobacilli* with anti-inflammatory effectors is significantly lower in KD children than in healthy controls and febrile cohorts^11^. It should be noted that these studies on the gut microbiota of KD patients were based on culture and polymerase chain reaction methods, which could not detect some low-abundance and non-cultivable pathogenic bacteria. Recently, a comparative metagenomic study with 28 KD children showed that *Streptococci* might contribute to the pathogenesis of KD with high abundance of genera *Rothia* and *Staphylococcus* in the acute phase, and high abundance of genera *Ruminococcus*, *Blautia*, *Faecalibacterium* and *Roseburia* in the non-acute phase^12^. However, healthy controls were not enrolled and a majority of the children received antibiotic treatment in this study, which may have affect the research outcomes.

Previous report demonstrated that an alteration in the intestinal microbiota composition contributed to several immune-mediated inflammatory diseases, such as inflammatory bowel disease, rheumatoid arthritis and multiple sclerosis^13^. Severe activation of the immune system and cascade release of inflammatory factors were regarded as the central features of KD^14^. The relationship between the composition of gut microbiota and inflammation in KD children has not been investigated yet. In this study, we sequenced the V3–V4 hypervariable regions of the 16S rRNA gene to gain a comprehensive profile of gut microbiota composition and to investigate its relationship with systemic inflammation in KD children.

## Materials and methods

### Study subjects

**Figure 1:**
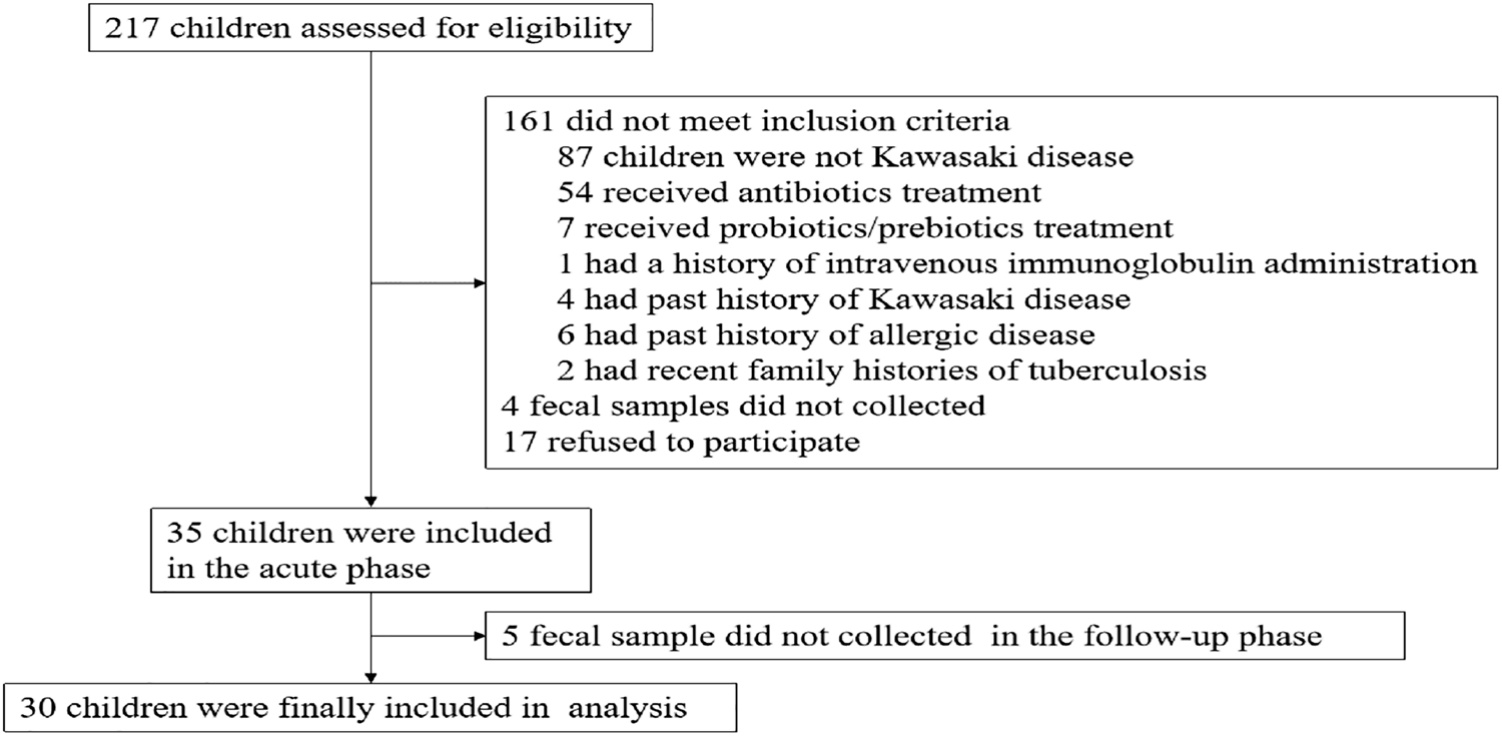
The flow diagram of children enrollment.

This study was performed in accordance with the ethical guidelines of and approved by the First Affiliated Hospital of Hunan Normal University Medical Ethics Committee. Written informed consent was obtained from parents or guardians of all participants before enrolment. The diagnosis of KD was established according to the guidelines of the American Heart Association (AHA)^15^. Children with a prolonged high fever lasting for at least five days were diagnosed with KD if they also presented with at least 4 of the following clinical symptoms: (1) diffuse oral cavity inflammation with fissure lips and strawberry tongue, (2) bilateral non-suppurative conjunctival injection, (3) polymorphous skin rashes, (4) indurative angioedema of the extremities, and (5) non-suppurative cervical lymphatic node with a diameter exceeding 1.5 cm. Children who suspected to being KD (had a high fever ≥ 39 °C and presented with at least 2 of the above clinical symptoms) were assessed on admission, from April 2017 to January 2019 (Fig. 1). Exclusion criteria were as follows: received antibiotics and probiotics/prebiotics treatment 3 months prior, previous immune modulating therapy, history of KD, concomitant with medical disorders (allergic disease, immunodeficiency, metabolic diseases and congenital diseases, et al.), fecal sample was not collected in the acute phase and/or non-acute phase. All KD cases were diagnosed by experienced clinicians. Those KD children were discharged following their recovery and followed up for 6 months. The healthy controls were recruited from children who had undergone a routine health visit at the First Affiliated Hospital of Hunan Normal University. Children with either bacterial or viral infection, chronic gastrointestinal disease, or had received antibiotics and probiotics/prebiotics treatment 3 months prior were excluded. The hospitalization duration was considered as the acute phase (K group); the non-acute phase was 6 months after onset (within 15 days beforehand or afterwards) (F group) and the healthy controls were described as the N group in Figures in this study.

### Serum sample collection and inflammatory biomarker assessment

Fasting venous blood samples (5 ml) were collected from both KD children on the admission and healthy controls on the day of physical examination, which were centrifuged at 4000* g* for 10 min at 20 °C. Serum samples were immediately frozen at − 80 °C. The serum levels of IL-2, IL-4, IL-6, IL-10, TNF-α and INF-γ were determined using flow cytometer (Cytomics FC 500, Beckman, USA). Briefly, the serum levels of IL-2, IL-4, IL-6, IL-10, TNF-α and INF-γ reacted with corresponding beads with different fluorescence that had been coated with capture specific antibodies. The quantitative determination of IL-2, IL-4, IL-6, IL-10, TNF-α and INF-γ were achieved by flow cytometry to detect different fluorescence intensities.

### Fecal samples collection and DNA extraction

Fresh fecal samples from children were collected in sterile plastic tubes upon admission but before antibiotic treatment. A second fecal sample was also collected from each KD child 6 months after disease onset (within 15 days beforehand or afterwards). Fecal samples of healthy controls were collected on the day of physical examination. All fecal samples were immediately transferred to the laboratory in an ice box and stored at − 80 °C within 15 min until DNA extraction. Total bacterial genomic DNA was extracted from the fecal samples using the DNA Isolation Kit (MoBio, Carlsbad, CA, United States) according to the manufacturer's instructions. The concentration and purity of bacterial genomic DNA were determined with a NanoDrop 2000 (Thermo Fisher Scientific, Waltham, USA) and the DNA integrity and quality were inspected by electrophoresis using a 1% agarose gel. All the genomic DNA was stored at − 20 °C prior to 16S rRNA gene sequencing.

### PCR amplification and sequencing

The V3-V4 hypervariable region of the 16S rRNA gene was amplified using the primers 338F (5′-ACTCCTACGGGAGGCAGCAG-3′) and 806R (5′-GGACTACHVGGGTWTCTAAT-3′) by the following amplification procedure: an initial denaturation at 98 °C for 2 min, 30 cycles (denaturation at 98 °C for 30 s, annealing at 50 °C for 30 s, elongation at 72 °C for 1 min), followed by a final extension at 72 °C for 5 min. The PCR reactions were carried out in a 50 μL volume containing 10 μl Buffer, 0.2 μL Q5 High-Fidelity DNA polymerase, 10 μL High GC Enhancer, 1 μ L dNTPs, 1.5 μL of each primer and 60 ng DNA template. The PCR products were recovered using a 1.8% agarose gel, purified using an MinElute PCR Purification Kit (Qiagen, Hilden, Germany) following the manufacturer’s instructions and quantified with NanoDrop2000. The purified amplicons were pooled in equal quantities. PE250 amplicon libraries were constructed and paired-end sequencing was performed according to the standard operating protocol of the Illumina HiSeq2500 platform (Illumina, San Diego, CA, USA) and index codes were added. The paired-end reads were then assembled using FLASH software and the UCHIME algorithm was used to screen for and remove putative chimeric sequences^16,17^. All paired-end reads were assigned to a sample according to their unique barcodes. Operational taxonomic units (OTUs) were clustered with a 97% identity threshold using the USEARCH software^18^. High-quality sequences with > 97% similarity was clustered into the same OTUs and taxonomy-based analyses were performed by classifying each 16S rRNA gene sequence using the RDP Classifier, and a cut-off value of 0.8 against the Silva 16S rRNA database (Release128, https://www.arb-silva.de) ^19^.

### Bioinformatics analysis

Mothur software was used to evaluate alpha diversity based on OTU level including indices to measure evenness (Simpson) and richness (ACE), Good’s coverage, and rarefaction curve analysis^20^. The difference in alpha diversity between groups was examined by Student's t-test. The principal coordinates analysis (PCoA) based on unweighted unifrac distances was performed to assess the beta diversity of the bacterial community using QIIME software and displayed by R software (Version 2.15.3)^21^. The linear discriminant analysis effect size (LEfSe) method combined with the standard tests for statistical significance (Kruskal–Wallis rank-sum test) with linear discriminant analyses (LDAs) was used to identify the effect of each differentially abundant taxon and to discriminate the metagenomic biomarkers^22^. An alpha significance level of 0.05 and an effect size threshold of 2 were used for discriminative microbial biomarkers. The functional composition of microbiota communities was predicted by PICRUSt from the 16S rRNA gene data^23^. Thereafter, a functional abundance spectrum of the Kyoto Encyclopedia of Genes and Genomes (KEGG) ortholog functional profile was acquired.

### Statistical analysis

Quantitative data were presented as mean ± standard deviation, and comparisons between KD and healthy children were performed through the Student's t-test or Mann–Whitney U test. Enumeration data were presented as proportions, and different groups were compared using chi-square tests. Spearman’s rank correlation was calculated to estimate the linear correlations between variables. The false discovery rate (FDR) was used for the P-value correction.

## Results

### Characteristics of study participants

We enrolled 30 KD children (average age 2.36 ± 1.39 years; male: female, 17:13) and 30 age- and sex-matched healthy children (average age 2.45 ± 1.27 years; sex, male: female, 16:14) from the First Affiliated Hospital of Hunan Normal University. All study participants are Chinese Han ethnicity. There were no significant differences in weight, height, delivery mode and feeding patterns between KD children and healthy controls. The levels of IL-2, IL-4, IL-6, IL-10, TNF-α and INF-γ in KD children were significantly elevated in the acute phase compared with healthy controls. A summary of related information is shown in Table 1.

**Table 1:** Characteristics of study participants.

	Acute KD children	Healthy controls	*P*-value
	n = 30	n = 30	
Sex (male/female)	17/13	16/14	0.80
Age (years)	2.36 ± 1.39	2.45 ± 1.27	0.79
Weight, kg	12.90 ± 3.21	13.07 ± 2.52	0.82
Height,cm	89.56 ± 11.76	90.26 ± 10.58	0.81
**Ethnicity**			
Han	30 (100%)	30 (100%)	
Non-Han	0	0	
**Delivery mode**			0.61
Cesarean section	56.67% (17)	50% (15)	
Natural birth	43.33% (13)	50% (15)	
**Feeding patterns**			0.49
Breastfeeding	80% (24)	86.67% (26)	
Artificial feeding	20% (6)	13.33% (4)	
Hospitalization (days)	10 (11–12)		
Resistant to IVIG treatment	2 (6.67%)		
Time from disease onset to diagnosis (days)	6 (5–7)		
Respiratory infection	4 (13.33%)		
**Diarrhoea/vomiting**	5 (16.67%)		
IL-2 (pg/ml)	11.83 ± 6.53	3.40 ± 1.61	< 0.001
IL-4 (pg/ml)	17.59 ± 14.03	1.36 ± 0.72	< 0.001
IL-6 (pg/ml)	101.88 ± 87.54	4.99 ± 2.08	< 0.001
IL-10 (pg/ml)	27.30 ± 16.35	4.21 ± 5.63	< 0.001
TNF-α (pg/ml)	7.64 ± 3.47	2.01 ± 1.05	< 0.001
IFN-γ (pg/ml)	37.43 ± 28.55	6.50 ± 2.81	< 0.001

*IVIG* intravenous immunoglobulin, *IL-2* interleukin-2, *IL-4* interleukin-4, *IL-6* interleukin-6, *IL-10* interleukin-10, *TNF-α* tumor necrosis factor α, *IFN-γ* interferon-γ. *P* < 0.05 is considered statistically significant.

### Differences of gut microbial diversity among acute KD children, healthy controls and non-acute KD children

**Figure 2:**
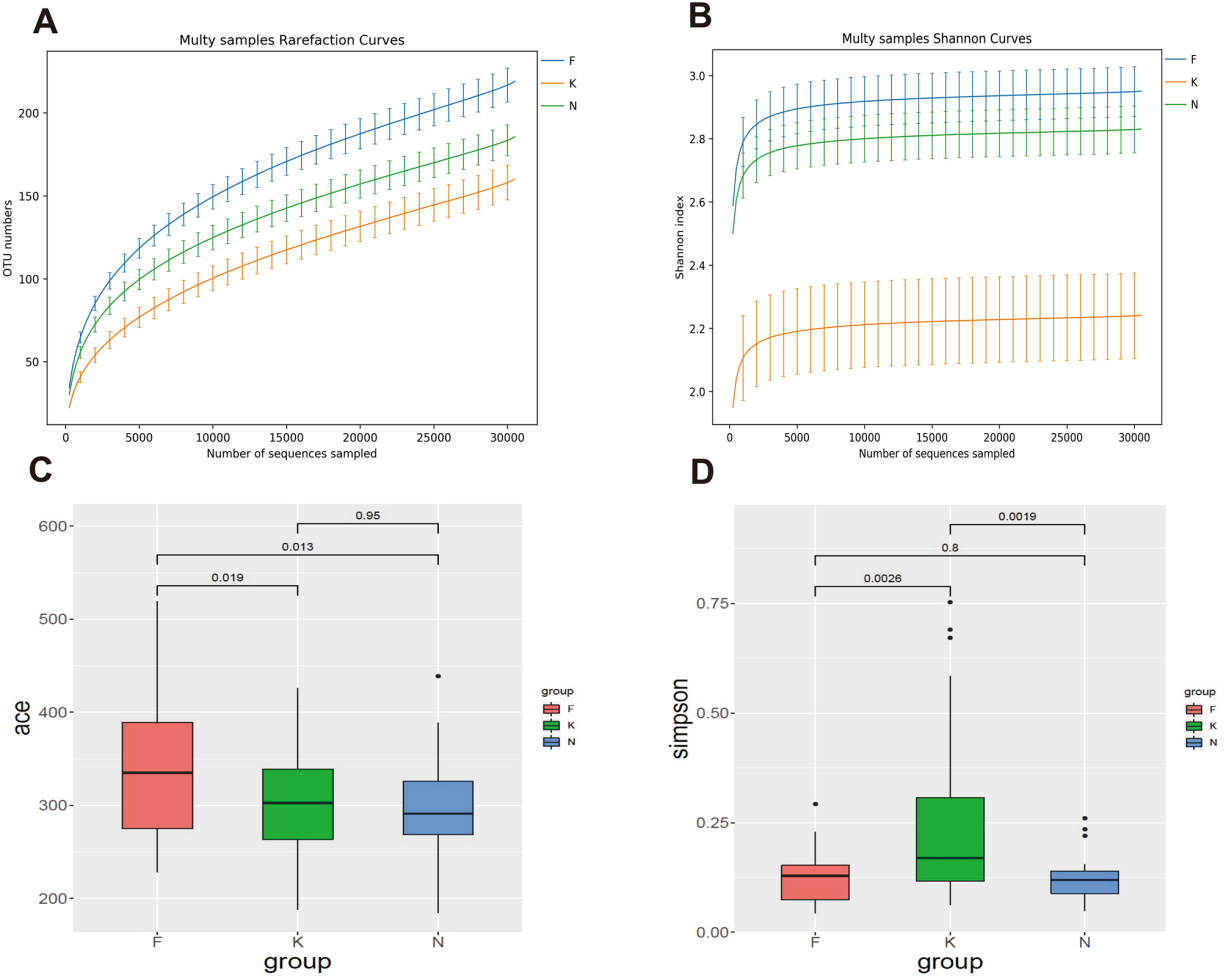
Alpha diversity analysis between acute KD children, non-acute KD children and healthy controls. (**A**) Rarefaction curve and shannon index curve (**B**) were used to estimate the sequencing depth (at a 97% similarity level) of the fecal microbiota between three groups. ACE index (**C**) revealed that non-acute KD children have higher bacterial richness than the acute KD children and healthy controls. Simpson index (**D**) revealed that acute KD children have a lower biodiversity than the non-acute KD children and healthy controls. *K* acute KD children, *F* non-acute KD children, *N* healthy controls.

**Figure 3:**
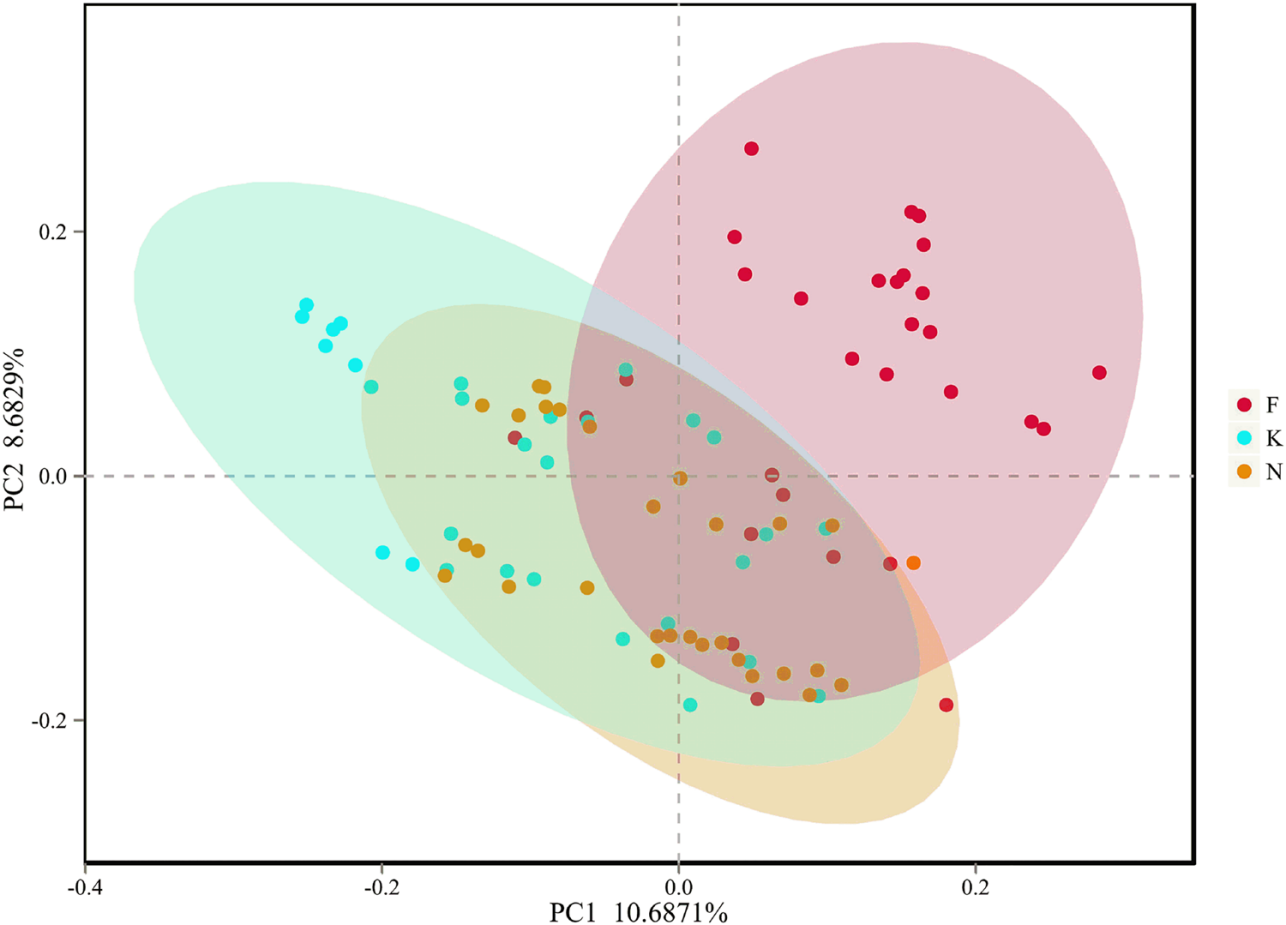
PCoA of bacterial beta diversity based on unweighted UniFrac distances revealed that the fecal microbiota of acute KD children was distinct from that of healthy controls and non-acute KD children (p = 0.001, PERMANOVA). *K* acute KD children, *F* non-acute KD children, *N* healthy controls.

This study obtained 6,804,869 raw sequences with a mean length of 416 base pairs from the 90 fecal samples. After quality trimming and chimera checking, totally 5,333,514 high-quality sequences remained with an average of 59,261 high-quality sequences per sample for downstream analysis. We obtained 1,131 OTUs in all samples of three groups: 803, 912, and 836 OTUs were identified in acute KD children, non-acute KD children and healthy controls respectively. This was obtained after clustering at a 97% similarity level. The information summary is shown in Table 2. Good’s coverage values were nearly 99% for all sequences in the three groups. The rarefaction curve showed slight slopes in the curve (Fig. 2A), indicating that new OTUs would be discovered if a greater sequencing depth was performed. Although the rarefaction curve approached an asymptote as the number of sequences increased, the Shannon index curve had already reached a plateau in all three groups (Fig. 2B). These results indicate that most fecal microbiota species were captured, providing a good sequencing depth for investigating the fecal microbiota in children. We calculated alpha diversity indices to evaluate the overall fecal microbiota richness and structural difference among these three groups. We observed that the microbiota richness of non-acute KD children was significantly increased compared to that of acute KD children and healthy controls. However, there were no significant differences between acute KD children and healthy controls as indicated by the ACE index (Fig. 2C). In contrast to the richness indices, the acute KD children had a lower biodiversity compared with healthy controls and non-acute KD children. There is no difference in microbial biodiversity between healthy controls and non-acute KD children, as indicated by the Simpson indices (Fig. 2D). This suggests that the gut microbiota of KD children is related to a loss in biodiversity. To evaluate the similarity of gut microbial communities between two groups, we detected the beta diversity by performing PCoA based on unweighted UniFrac distances, which revealed that the fecal microbiota of acute KD children was distinct from that of healthy controls and non-acute KD children (*p* = 0.001, PERMANOVA) (Fig. 3). The separation trend of the non-acute phase and the healthy controls is more significant than that of the acute phase and the healthy controls (Fig. 3). Due to lack of specific laboratory diagnostic indicators, the diagnosis of KD is based on an assessment of nonspecific clinical symptoms, which still remains a major challenge^24^. The mean of time from disease onset to diagnosis of KD was 6 days (range: 5–8 days) in this study. KD children were characterized by a prolonged high fever, which is usually misdiagnosed as an infection before the correct diagnosis. The majority of KD children therefore received antibiotic therapy empirically, which may exert a long-term effect on the composition of the gut microbiota.

**Table 2:** Fecal microbiota community indices between K group, F group and H group.

Group	OTUs	Good’s	Richness index	Diversity index
			ACE	Simpson
K	803	0.9988	298.80 ± 10.77	0.24 ± 0.04
F	912	0.9985	333.61 ± 12.61	0.12 ± 0.01
N	836	0.9988	297.81 ± 9.73	0.12 ± 0.01

*K* acute KD children, *F* non-acute KD children, *N* healthy controls.

### Differences in microbiota composition between acute KD children, healthy controls and non-acute KD children

**Figure 4:**
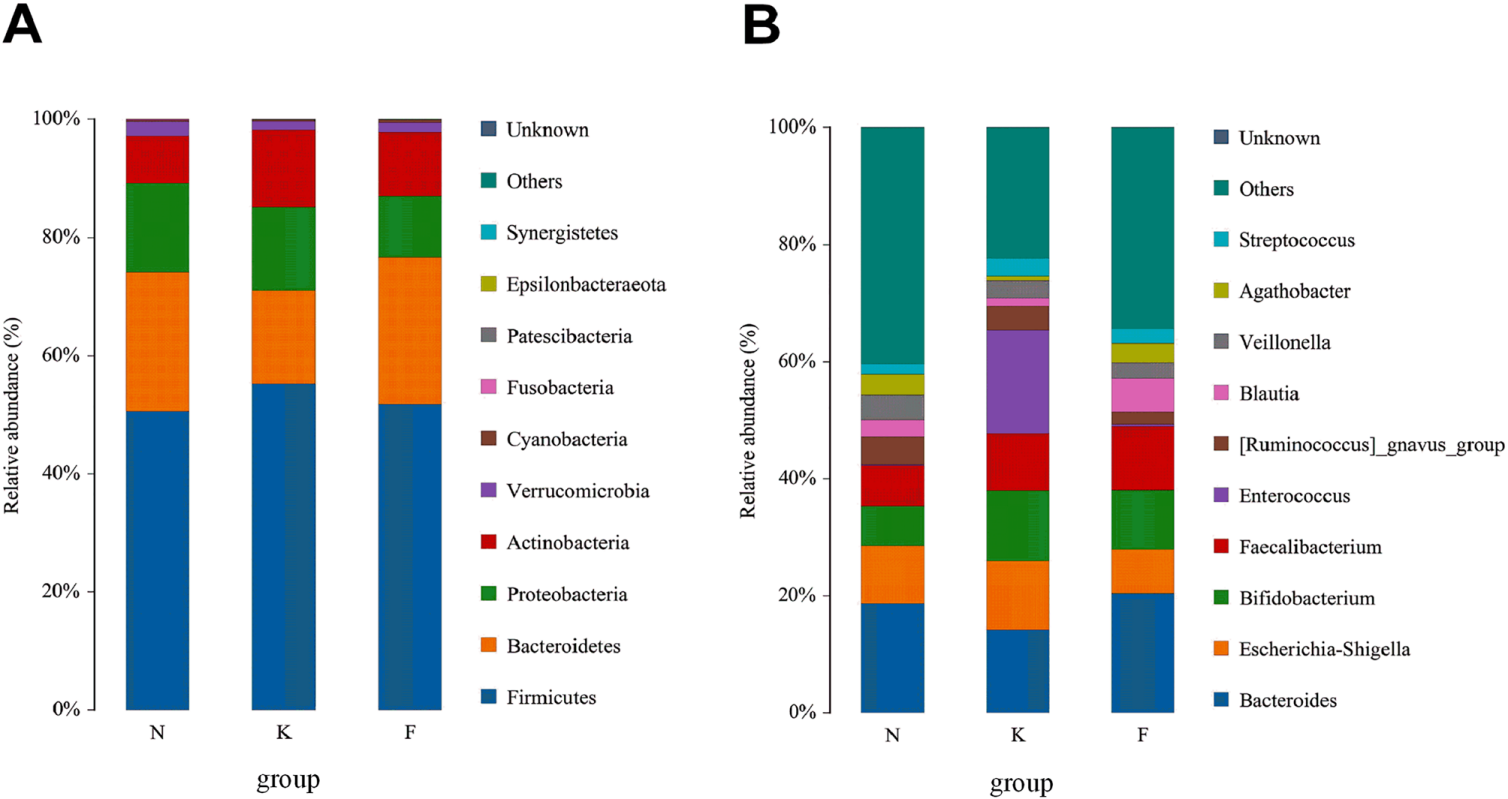
Distribution of gut microbiome between acute KD children, non-acute KD children and healthy controls at the levels of phylum and genus. (**A**) *Firmicutes*, *Bacteroidetes*, *Proteobacteria* and *Actinobacteria* were the four major phyla in the three groups. (**B**) *Enterococcus*, *Bacteroides*, *Bifidobacterium* and *Escherichia-Shigella* were the predominant genera in acute KD children; *Bacteroides*, *Faecalibacterium*, *Bifidobacterium* and *Escherichia-Shigella* were the predominant genera in non-acute KD children; *Bacteroides*, *Escherichia-Shigella*, *Faecalibacterium* and *Bifidobacterium* were predominant genera in healthy controls. *K* acute KD children, *F* non-acute KD children, *N* healthy controls.

**Figure 5:**
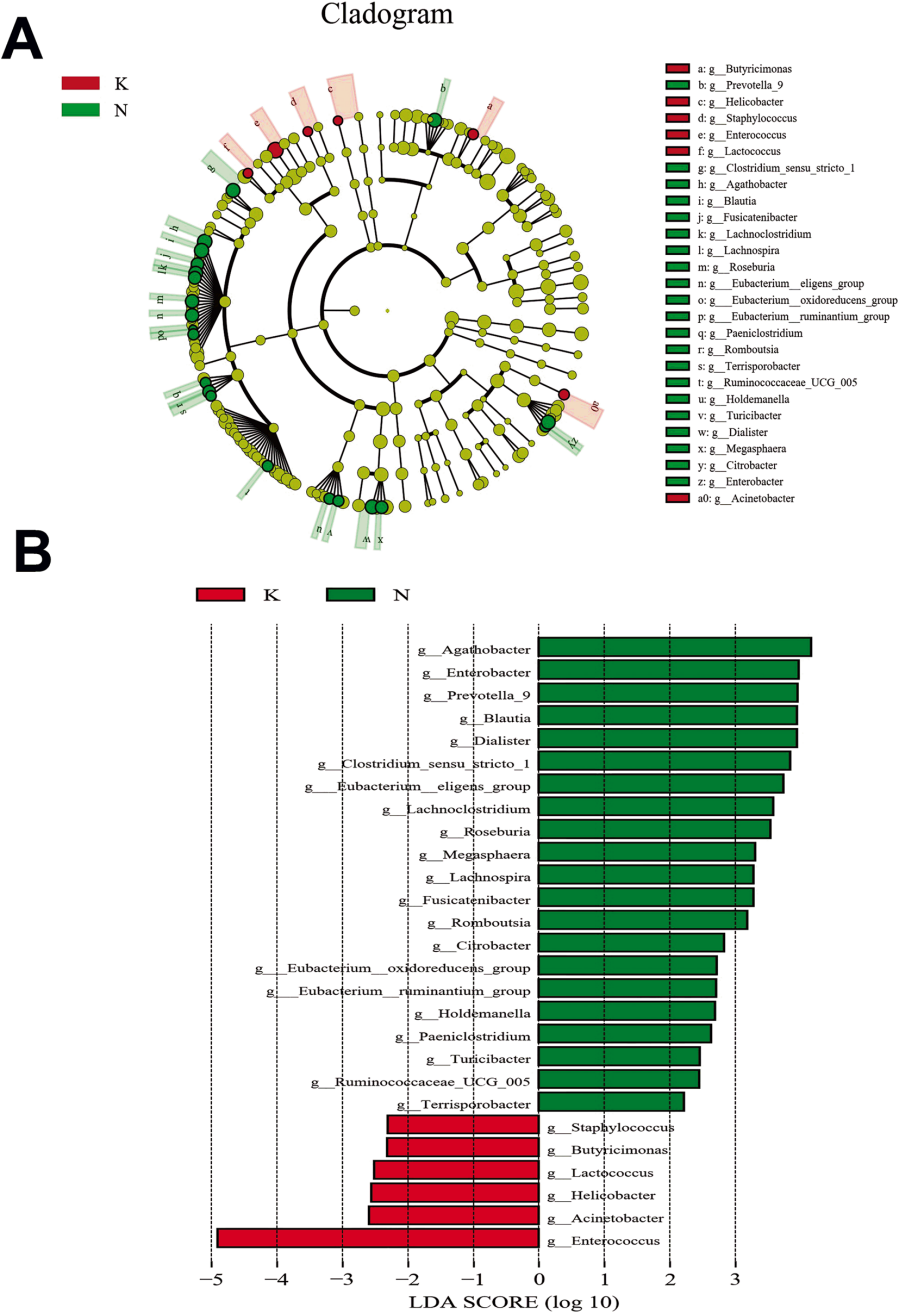
LEfSe identified the most differentially abundant taxons between acute KD children and healthy controls. (**A**) Taxonomic cladogram obtained from LEfSe analysis of 16S sequences. (Red) tax enriched in acute KD children; (green) tax enriched in healthy controls. The brightness of each dot is proportional to its effect size. (**B**) Healthy controls enriched taxa are indicated with a positive LDA score (green) and taxa enriched in acute KD children have a negative score (red). The LDA score indicates the effect size and rank of differentially abundant taxon. The threshold for the logarithmic LDA score was 2.0. *K* acute KD children, *N* healthy controls.

**Figure 6:**
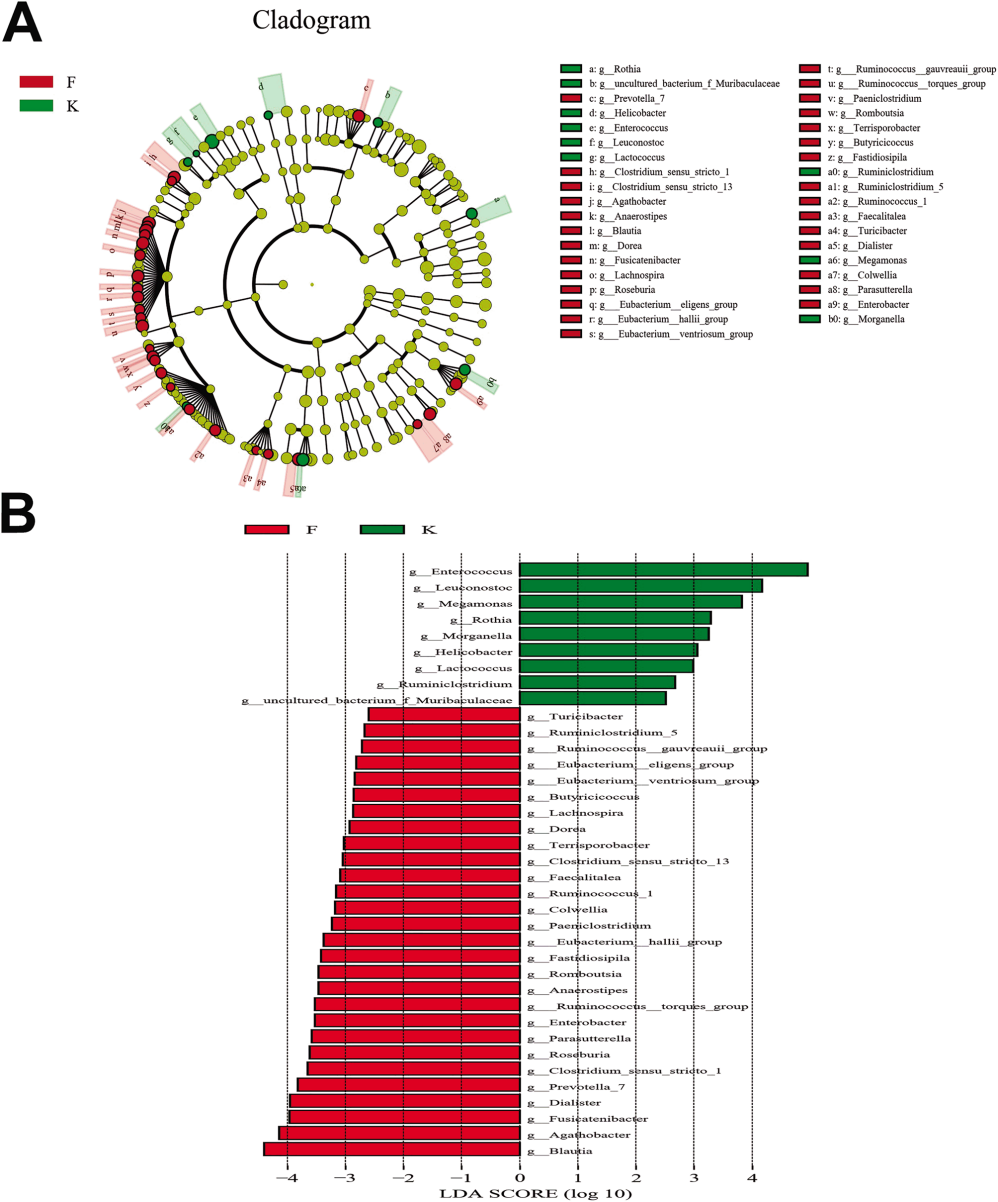
LEfSe identified the most differentially abundant taxons between acute KD children and non-acute KD children. (**A**) Taxonomic cladogram obtained from LEfSe analysis of 16S sequences. (Red) tax enriched in non-acute KD children; (green) tax enriched in acute KD children. The brightness of each dot is proportional to its effect size. (**B**) Enriched genera in the acute KD children were indicated by a positive LDA score (green); enriched genera in the non-acute KD children were indicated with a negative score (red). The LDA score indicates the effect size and rank of each differentially abundant taxon. The threshold for the logarithmic LDA score was 2.0. *K* acute KD children, *F* non-acute KD children.

The taxonomy of fecal microbiota was assessed via taxon dependent analysis using the RDP classifier. *Firmicutes*, *Bacteroidetes*, *Proteobacteria* and *Actinobacteria* were the four major phyla in fecal samples of three groups, accounting for 97% of all sequences (Fig. 4A). However, we observed no significant difference in microbial composition between the three groups at phyla level. At genus level, *Enterococcus* (17.62%), *Bacteroides* (14.22%), *Bifidobacterium* (11.97%), and *Escherichia-Shigella* (11.80%) were the most predominant genera in fecal samples of acute KD children, whereas *Bacteroides* (20.41%), *Faecalibacterium* (10.93%), *Bifidobacterium* (10.12%) and *Escherichia-Shigella* (7.51%) were the most predominant genera in fecal samples of non-acute KD children. In comparison, predominant genera in healthy controls were *Bacteroides* (18.70%), *Escherichia-Shigella* (9.82%), *Faecalibacterium* (6.93%) and *Bifidobacterium* (6.86%) (Fig. 4B). To determine the potential bacterial biomarkers that drove the differentiation of the microbiota between acute KD children, healthy controls and non-acute KD children, we used the LEfSe analysis at genus level (LDA score > 2, p < 0.05). *Enterococcus*, *Acinetobacter*, *Helicobacter*, *Lactococcus*, *Staphylococcus* and *Butyricimonas* were significantly enriched in acute KD children, while SCFA producing microbiota such as *Prevotella*, *Dialister*, *Clostridium*, *Eubacterium, Roseburia* and *Megasphaera* were significantly reduced in acute KD children (Fig. 5). We also investigated the alteration in the gut microbiota composition between the acute and non-acute phases of KD using LEfSe, and observed that *Enterococcus*, *Leuconostoc*, *Megamonas*, *Helicobacter*, *Morganella* and *Lactococcus* were significantly increased in acute KD children, whereas the SCFA producing microbiota including *Blautia*, *Prevotella*, *Dialister*, *Clostridium*, *Roseburia*, *Anaerostipes*, *Ruminococcus*, and *Dorea* were significantly enriched in non-acute KD children (Fig. 6).

### Functional capability analysis

**Figure 7:**
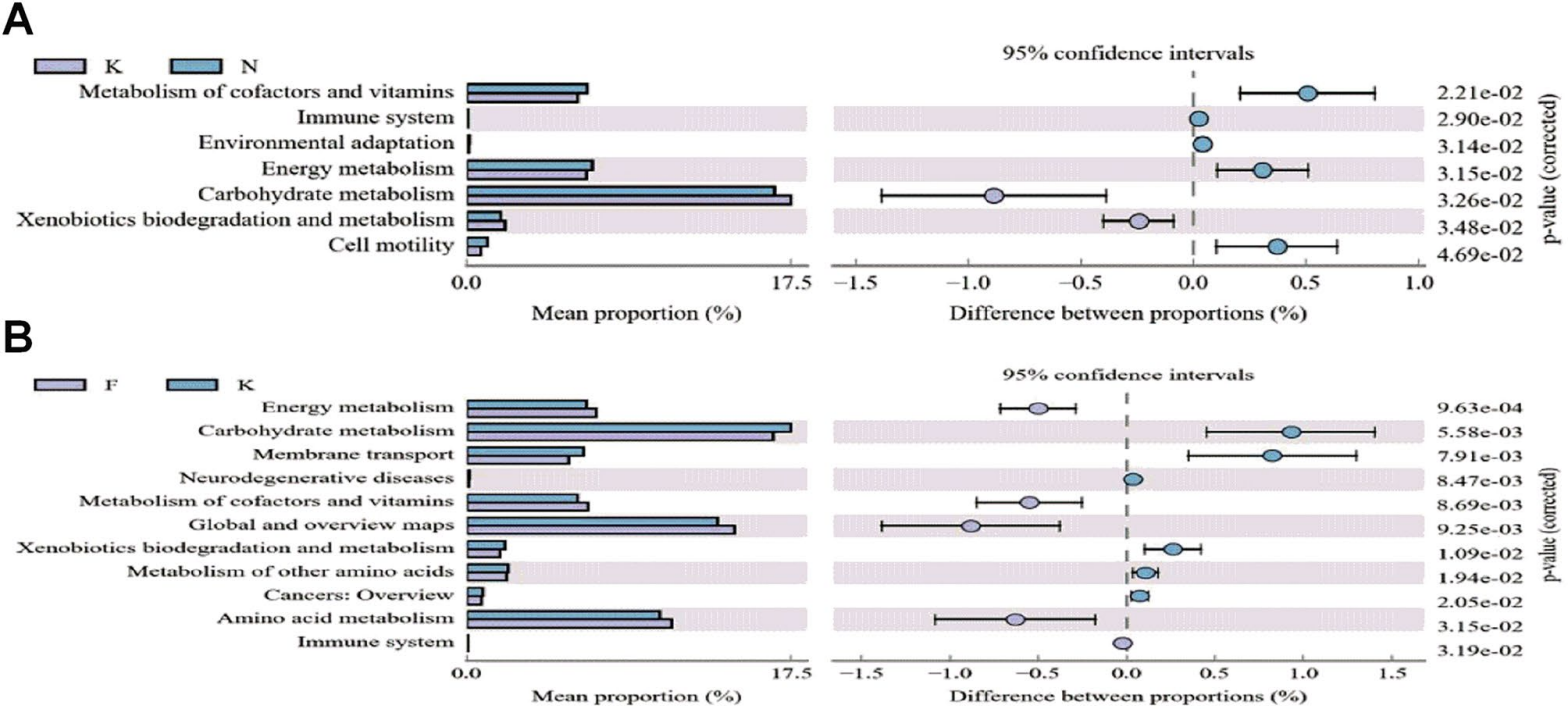
Functional capability analysis based on the mean abundances of KEGG pathways. (**A**) Functional differences between acute KD children and healthy controls. Pathways that were more abundant in healthy controls are on the positive side. Pathways that were more abundant in acute KD children are on the negative side (orange circle with 95% confidence interval). (**B**) Functional differences between acute KD children and non-acute KD children. Pathways that were more abundant in acute KD children are on the positive side. Pathways that were more abundant in non-acute KD children are on the negative side.

To clarify how normal biological functions in KD children may be affected, the functional gene composition of the fecal microbiota in above samples was analyzed from the 16S rRNA gene data with PICRUSt. In acute KD children, the functional genes for carbohydrate metabolism, xenobiotics biodegradation and metabolism were highly enriched in comparison with those of healthy controls. Additionally, the metabolism of cofactors and vitamins, environmental adaptation, energy metabolism and cell motility functional genes were significantly lowered in acute KD children (Fig. 7A). Carbohydrate metabolism and xenobiotics biodegradation and metabolism in non-acute KD children were significantly lower than in acute KD children (Fig. 7B).

### Correlation of the gut microbiota with inflammatory markers

The biomarkers of IL-2, IL-4, IL-6, IL-10, TNF-α, and INF-γ were selected to assess the systemic inflammation in acute KD children. We observed that several bacterial species were correlated with inflammatory factors (shown in Table 3). *Enterococcus and Helicobacter* were correlated positively with IL-6 (r = 0.39, *p* = 0.04; r = 0.37, *p* = 0.00, respectively).

**Table 3:** Correlation between the abundance of specific taxa and serum inflammatory biomarkers.

	IL-2		IL-4		IL-6		IL-10		TNF-α		IFN-γ	
	r	p	r	p	r	p	r	p	r	p	r	p
Enterococcus	0.39	0.20	0.16	0.79	0.39	**0.04**	0.15	0.78	0.06	0.98	0.31	0.41
Acinetobacter	0.13	0.88	0.06	0.98	0.09	0.98	0.03	0.98	− 0.07	0.98	0.05	0.98
Helicobacter	0.24	0.63	0.03	0.98	0.37	**0.00**	0.14	0.78	− 0.04	0.98	0.23	0.64
Lactococcus	0.12	0.95	0.09	0.98	0.24	0.64	0.22	0.54	− 0.06	0.98	0.14	0.86
Staphylococcus	0.09	0.98	− 0.07	0.98	0.07	0.98	− 0.04	0.98	− 0.01	0.98	0.10	0.98
Butyricimonas	− 0.01	1	− 0.06	0.98	− 0.02	0.98	0.02	0.98	0.03	0.98	− 0.07	0.98
Lachnospiraceae_UCG-004	− 0.37	0.20	− 0.29	0.32	− 0.29	0.40	− 0.01	0.98	− 0.06	0.98	− 0.18	0.79
Rothia	0.37	0.20	0.21	0.54	0.38	0.20	0.12	0.95	0.14	0.86	0.30	0.32
Bacteroides	− 0.31	0.20	− 0.18	0.71	− 0.26	0.41	− 0.03	0.98	− 0.09	0.98	− 0.15	0.84
Tyzzerella	0.31	0.26	0.24	0.50	− 0.06	0.98	0.03	0.98	0.26	0.41	0.11	0.98
Coprobacillus	0.28	0.26	0.33	0.20	0.14	0.86	0.23	0.41	0.16	0.71	0.20	0.71
Escherichia-Shigella	− 0.31	0.26	− 0.07	0.98	− 0.16	0.79	− 0.09	0.98	− 0.23	0.52	− 0.02	0.98
Alloprevotella	0.28	0.41	0.05	0.98	0.34	0.26	0.21	0.54	− 0.01	1	0.23	0.53
Clostridium_sensu_stricto_1	− 0.23	0.62	− 0.09	0.98	− 0.15	0.86	− 0.23	0.58	− 0.38	0.2	− 0.02	0.98
[Eubacterium]_eligens_group	− 0.29	0.30	− 0.29	0.41	− 0.13	0.95	− 0.04	0.98	− 0.06	0.98	− 0.33	0.26

*P* < 0.05 is considered statistically significant.

## Discussion

Increasing evidence supported the notion that alterations in gut microbiota contribute to the pathogenesis of KD. The composition of gut microbiota was not fully investigated and the linkage between gut microbiota changes and systemic inflammation have not been determined as well. In this study, we investigated the composition of gut microbiota in KD children using 16S rRNA gene sequencing of fecal samples and assess its relationship with systemic inflammation. We observed that acute KD children were characterized by a dysbiotic gut microbiota with reduced microbial diversity, as well as an alteration in the composition of the gut microbiota. Several altered abundances of the microbiota genera were correlated with systemic inflammation. Our results provide novel insights into the complex host-microbiota interaction in KD.

A recent comparative metagenomic analysis showed that *Streptococci* might contribute to KD pathogenesis and that the relative abundance of *Rothia* and *Staphylococcus* were highly enriched in the acute phase, whereas the relative abundance of *Ruminococcus*, *Blautia*, *Faecalibacterium*, and *Roseburia* were significantly increased in the non-acute phase^12^. Our findings on the relative abundance of *Ruminococcus*, *Blautia* and *Roseburia* in the non-acute phase were comparable to these previous reports, however, we found that *Enterococcus*, *Leuconostoc*, *Megamonas*, *Helicobacter*, *Morganella* and *Lactococcus* were significantly enriched in the acute phase, which was inconsistent with their results. The majority of acute KD children in their study received cephalosporin antibiotic treatment empirically and this has a powerful effect of reducing gram-positive cocci including *Enterococcus* and *Lactococcus.* Therefore, their results may reflect the effects of the antibiotic treatment. Moreover, the composition of gut microbiota is affected by dietary habits, genetics, antibiotics, hygiene conditions and other host-associated factors^25^. The gut microbiota are also different among individuals from different races and ethnicities^26^. The individuals’ race in our study was different from theirs, which may account for this result inconsistency.

Healthy controls were also enrolled in our study to explore significant changes in the fecal microbiota composition and explore microbiota biomarkers for acute KD children. LEfSe analysis showed that *Enterococcus*, *Acinetobacter*, *Helicobacter*, *Lactococcus*, *Staphylococcus* and *Butyricimonas* were significantly enriched in acute KD children. *Enterococci* are normal gut microbes in humans and *Enterococcus* pathogens commonly cause nosocomial infections affecting the bloodstream, urinary tract, peritoneum and respiratory tract^27,28^. *Enterococcus* usually has high biofilm formation ability and these biofilms can produce a large variety of biologically active molecules including superantigens, and also induce a strong inflammatory response^29^. These are parts of the pathogenesis of KD^30^. Consistent with previous studies, we found that the relative *Staphylococcus* abundance was increased in acute KD children. Yamashiro et al. showed that the bacterial species of the jejunum in KD children were predominantly gram-positive cocci, of which *Streptococci* and *Staphylococci* were isolated from the children^10^. Nagata et al. also identified *Streptococcus* and *Staphylococcus* with superantigenic properties in KD children^9^. A study related to the mucosal surface colonization of acute KD patients showed that toxic shock syndrome toxin 1-producing strains of *Staphylococcus* aureus are associated with the etiology of acute KD^31^. Our results confirmed the role of *Staphylococcus* in the pathogenesis of KD. The *Lactococcus* genus, a member of *Streptococcaceae* family, is a kind of Lactic Acid Bacteria (LAB) that produces lactic acid from the fermentation of carbohydrates^32^. *Lactococcus* is not normally part of the gut microbiome and is considered as an opportunistic pathogen that exists in the vast majority of fermented foods^33^. It was reported that *Lactococcus* could result in systemic human infections such as bacteremia, osteomyelitis, endocarditis, and peritonitis^34^. This study identify for the first time the relationship between *Lactococcus* and KD pathogenesis. *Lactococcus* may be involved in the pathogenesis of KD as an opportunistic pathogen due to dysbiosis of the gut microbiota. Furthermore, we found that *Acinetobacter* was associated with KD, the *Acinetobacter* may produce HSP60 with a molecular structure partially similar to that of human HSP60, which induces the production of autoreactive B cells and cytotoxic T cells that contribute to KD development^9,35^. In addition, we observed that *Helicobacter* and *Butyricimonas* were involved in the pathogenesis of KD, which has never been reported previously. However, the roles of *Helicobacter* and *Butyricimonas* in the pathogenesis of KD are still not clear.

We also found that the SCFA-producing microbiota such as *Prevotella*, *Dialister*, *Clostridium*, *Eubacterium, Roseburia* and *Megasphaera* were significantly reduced in acute KD children compared with those of healthy controls. Moreover, the SCFA-producing microbiota including *Blautia*, *Prevotella*, *Dialister*, *Clostridium*, *Roseburia*, *Anaerostipes*, *Ruminococcus*, and *Dorea* were significantly enriched in non-acute KD children. Gut microbiota have anti-inflammatory effects and can metabolize dietary fiber and nondigestible carbohydrates to produce SCFAs including acetate, butyrate and propionate^36^. Reductions in butyrate-producing microbiota may exacerbate some forms of IBD^37,38^. It was reported that reduction in SCFA producing gut microbiota such as *Akkermansia*, *Christensenellaceae*, *Clostridium*, and *Odoribacter* can enhance systemic inflammation and accelerate atherogenesis in Ldlr − / − mice^39^. Consistent with these above studies, we also observed a reduction of SCFAs producing microbiota in acute KD children, suggesting that depletion of this SCFA producing microbiota is closely related to KD pathogenesis.

The strong activation of immune system and cascade release of inflammatory factors were the central features of KD. A variety of pro-inflammatory and anti-inflammatory factors such as IL-6, IL-10, TNF-α, and INF-γ were significantly increased in KD children before treatment^40^. Consistent with this study, our results showed that the levels of IL-2, IL-4, IL-6, IL-10, TNF-α and INF-γ were significantly elevated in acute KD children compared with healthy controls. The correlation analysis showed that *Enterococcus and Helicobacter* were correlated positively with IL-6 (r = 0.39, *p* = 0.04; r = 0.37, *p* = 0.00, respectively). Indeed, *Enterococcus* strains can perpetuate and amplify the inflammatory response in IL-10-/- mice and *Helicobacter pylori* may lead to recruitment and activation of various T cells, including mucosal-associated invariant T cells and T helper-17, which magnify and maintain inflammation^41,42^. Therefore, we speculated that dysbiosis in the composition of gut microbiota may participate in the pathogenesis of KD by amplifying systemic inflammation. However, further studies would need to be conducted in this regard.

We observed significant differences in the presence of microbial community functional abundance between acute KD children and healthy controls. Carbohydrate metabolism was significantly enhanced in acute KD children and this may be attributed to *Enterococcus*, whose presence was significantly high in acute KD children in our study. Non-motile *Enterococcus faecalis* can strengthen its invasive toxicity and switch from commensal to pathogen by utilizing carbohydrate metabolism such as RpiA-GlnA-EpaX metabolic axis^43^. In the gut micro-ecology of acute KD children, the metabolism of cofactors and vitamins, immune system, environmental adaptation, energy metabolism and cell motility functions were weakened, which may directly affect the colonization and reproduction of gut microbiota.

## Conclusion

In the present study, we unveiled that the increase in the relative abundance of *Enterococcus*, *Acinetobacter*, *Helicobacter*, *Lactococcus*, *Staphylococcus* and *Butyricimonas* with a reduction in short chain fatty acids (SCFA) producing microbiota promote the gut microbiota dysbiosis associated with KD, which were never reported in previous studies. Additionally, we observed that disturbance in the composition of gut microbiota was associated with systemic inflammation in KD children. Our results provided new insights into the etiology of KD, which will deepen our understanding of its pathogenesis and facilitate the development of new therapeutic approaches.

## Data Availability

The raw data are available at the US National Center for Biotechnology Information (NCBI) (https://www.ncbi.nlm.nih.gov/sra/PRJNA595748).
